# Inhibitory Effect of GRAS Essential Oils and Plant Extracts on the Growth of *Aspergillus westerdijkiae* and *Aspergillus carbonarius* Strains

**DOI:** 10.3390/molecules27196422

**Published:** 2022-09-29

**Authors:** Mariana Paiva Rodrigues, Águida Aparecida de Oliveira, Gabriela Lago Biscoto, Priscila Natália Pinto, Raul Roque de Souza Dias, Lauranne Alves Salvato, Luiz Antonio Moura Keller, Lilia Reneé Cavaglieri, Carlos Alberto da Rocha Rosa, Kelly Moura Keller

**Affiliations:** 1 Programa de Pós-Graduação em Ciência Animal, Escola de Veterinária, Universidade Federal de Minas Gerais, Belo Horizonte 30270-901, Brazil; 2 Departamento de Microbiologia e Imunologia Veterinária, Instituto de Veterinária, Universidade Federal Rural do Rio de Janeiro, Seropédica, Rio de Janeiro 23890-000, Brazil; 3 Departamento de Medicina Veterinária Preventiva, Escola de veterinária, Universidade Federal de Minas Gerais, Belo Horizonte 30123-970, Brazil; 4 Departamento de Zootecnia e Desenvolvimento Agrossocioambiental Sustentável, Faculdade de Veterinária, Universidade Federal Fluminense, Niterói, Rio de Janeiro 24230-340, Brazil; 5 Consejo Nacional de Investigaciones Científicas y Técnicas, Departamento de Microbiología e Inmunología, Facultad de Ciencias Exactas, Físico Químicas y Naturales, Universidad Nacional de Río Cuarto, Río Cuarto X5804BYA, Córdoba, Argentina

**Keywords:** fungal contamination, natural products, mycotoxins, ochratoxin A, GRAS

## Abstract

The effect of essential oils (obtained using hydrodistillation) and plant extracts (ethanolic, aqueous, and hexanic extractions) of 10 different plants cultivated in Brazil were tested using the diffusion agar method, with the objective of evaluating the inhibitory effect of the oils and extracts on the mycelial growth of *Aspergillus westerdijkiae* NRRL 3174 and *A. carbonarius* RC 2054 (UNRC). Of the 40 essential oils and plant extracts analyzed, oregano essential oil and plant extract, rosemary essential oil, and the clove ethanolic extract were the best choice to obtain the growth parameters (radial growth rates (mm day^−1^) and lag phase (h)) due the good results presented and the volume of oil/extract obtained. Comparing all the essential oils and plant extracts that were tested for growth parameters, the best results were obtained for the clove ethanolic extract for both strains assayed. These results demonstrated an outstanding potential use of some of these products in prevention of fungal contamination in food. However, further studies need to be conducted to determine the ability of these oils and extracts to inhibit or reduce ochratoxin A production.

## 1. Introduction

Fungi are usual contaminants of foodstuffs. This contamination can lead to several economic losses, by deteriorating and reducing the nutritional value of the food. In addition, some fungal species have the potential to produce mycotoxins, which are secondary toxic metabolites [1,2].

Members of *Aspergillus* Section *Circumdati* (*A. ochraceus* group) are widely distributed and have already been isolated from different substrates, such as coffee, sorghum, soil, peanuts, grapes, air, house dust, and even from scalp lesions of humans [3]. Some species of this section have an important positive economical role, they participate in the biocatalysis of progesterone [4] and in the production of potentially antifungal compounds [5]. However, some species are known as relevant animal and human pathogens and mycotoxins producers. From the 28 species already identified by polyphasic taxonomy by Visagie et al. (2014) [6], 27 showed at least a trace production of ochratoxin A (OTA), penicillic acid, xanthomegnin, viomellein, or vioxanthin. The same study also showed that at least 20 species were able to produce OTA, and 5 species (*A. occultus*, *A. pulvericola*, *A. roseoglobulosus*, *A. steynii*, and *A. westerdijkiae*) exhibit strong OTA production. This proves the great relevance of species of section *Circumdati* in mycotoxin production [6]. However, some species of other *Aspergillus* sections are also OTA producers [7].

Members of *Aspergillus* section *Nigri* are important food contaminants, and some species have biotechnological applications, e.g., *A. niger* [8]. Although the classification and identification of the species are difficult, some had already been identified from different origins, e.g., coffee, pepper, grapes, water, beverage, soil, leaves, paper, sand, and different continents [9]. Among these species, *A. carbonarius*, *A. lacticoffeatus*, *A. sclerotioniger*, and *A. niger* play a relevant role in OTA production [10]. 

OTA is a potent nephrotoxin, which has already been correlated to the Balkan endemic nephropathy and urinary tumors [11], being classified by the International Agency for Research on Cancer (IARC) as a possible human carcinogen (group 2B) based on sufficient evidence of carcinogenicity for animals and inadequate evidence in humans [12]. Furthermore, OTA can show other toxic effects such as gastro-intestinal tract lesions, immunosuppression, genotoxicity, and cardiac and hepatic injuries and toxicity [13,14,15]. Due to all the losses caused by fungal contamination and the potential toxicity of the secondary metabolites, it is important to prevent, control, and reduce fungal growth in human and animal feed, especially with the use of generally recognized as safe substances (GRAS).

Essential oils (EOs) and plant extracts (PEs) are composed of aromatic volatile and non-volatile compounds of plants’ secondary metabolism, which are extracted from different parts of innumerous plants, such as leaves, barks, flowers, and seeds [16,17]. EOs have a potential antimicrobial activity, lipophilic tendencies, and low molecular weight; these characteristics allow them to quickly penetrate the cell membranes. In addition, they have also been recognized as antiviral and anthelmintic substances [16,18,19,20,21,22]. However, the antifungal effects of several essential oils are still not well described for different fungal species. Some studies had already analyzed the EO effects against some pathogenic fungal genera and some mycotoxigenic genera, such as *Fusarium* spp. and *Penicillium* spp. [16,23,24,25,26,27,28]. Among the mycotoxigenic species, the effects of innumerous EOs have already been described against the growth of *Aspergillus flavus*, *A. parasiticus*, and *A. niger* [18,23,24,26,27,29,30,31,32,33,34,35,36,37,38]. However, only a study comprising the antifungal effects of oregano (*Origanum vulgare*), mint (*Menta arvensis*), basil (*Ocimum basilicum*), sage (*Salvia officinalis*), and coriander (*Coriandrum sativum*) was conducted against *A. westerdijkiae* species [39]. Even though there are a few studies on the antifungal effects of cinnamon (*Cinnamomum zeylanicum*), clove (*Eugenia caryophyllata*), oregano (*Origanum vulgare*), and rosemary (*Rosmarinus officinalis*), against the growth of *A. carbonarius*, this study presents some innovative results about the antifungal potential of these and other EO and plant extracts, used in different concentrations and obtained using different methods [26,40,41,42,43].

Due to the potential antifungal effects and the lack of studies comprising some essential oils against the growth of *A. westerdijkiae* and *A. carbonarius* strains, this study aimed to evaluate the inhibitory effect of EOs and PEs extracted from 10 different plants recognized as GRAS by the Food and Drug Administration (FDA) by hydrodistillation (EO), hexanic (EH), aqueous (AE), and ethanolic extraction (EE) [44].

## 2. Results

### 2.1. Initial Screening

The diameter of the inhibition zone of fungal growth, of the two strains studied, caused by 50 μL of essential oil or plant extract was measured in mm on the fifth day of incubation at 25 °C. The results for *A. westerdijkiae* and *A. carbonarius* are shown in Table 1 and Table 2, respectively. Cinnamon essential oil showed the best inhibitory effect among the other plant EOs on both strains. The plant extracts obtained from hexanic extraction (EH) of all plants showed a complete inhibition on both strains. However, the low quantity obtained from some EO, EH, and EE made it impossible to conduct further tests. Furthermore, it is important to highlight that the aqueous extract of all plants did not show any inhibitory effect on both *Aspergillus* species tested. Clove showed the best result in both strains for ethanolic extracts. It is important to highlight that during this initial screening the interaction between the solvents used and both strains were analyzed, and no inhibition was observed.

### 2.2. Effect of the Essential Oils and Plant Extract on Lag Phase and Growth Rate 

The oregano essential oil obtained using hydrodistillation and the oregano plant extract obtained using hexanic extraction, the rosemary essential oil (obtained using hydrodistillation), and the clove ethanolic extract were chosen to obtain the growth parameters at concentrations of 0, 50, 100, 150, 300, and 600 mg kg^−1^ for both fungal strains on Yeast Extract Agar (YES). Comparing all three methods of extraction on both strains, the best inhibitory effect and increased lag phase result was for the clove ethanolic extract, however, the best hydrodistilled essential oil result was obtained from oregano EO.

The use of rosemary EO resulted in a growth rate that ranged from 5.3 to 6.1 mm day^−1^, and a lag phase between 25 and 31 h, for *A. westerdijkiae* (Table 3). There were no statistically significant differences in the values of growth rate except for the 100 mg kg^−1^ concentration (*p* < 0.05), however, for the lag phase values only the 600 mg kg^−1^ concentration was statistically similar to the control.

For *A. carbonarius*, the growth rate values ranged from 10.0 to 10.8 mm day^−1^ and the lag phase varied from 25 to 28 h with rosemary EO (Table 4). There were no statistically significant differences in the values of growth rate and lag phase (*p* < 0.05).

The *A. westerdijkiae* growth rate using oregano EO ranged from 1.3 to 5.6 mm day^−1^ and the lag phase was between 33 and 88 h (Table 5). The *A. carbonarius* growth rate was from 2.8 to 11.0 mm day^−1^ and the lag phase was between 33 and 53 h (Table 5). In all concentrations the growth rate and lag phase were statistically different to the control for both strains, except for *A. westerdijkiae* lag phase at the concentration of 150 mg kg^−1^ (Table 5).

The use of oregano EH on *A. westerdijkiae* resulted in a growth rate that varied from 5.3 to 6.2 mm day^−1^ and a lag phase between 27 and 35 h (Table 6). For the growth rate values, only the concentrations of 150 and 300 mg kg^−1^ were statistically different to the control (*p* < 0.05) as well as the 150 mg kg^−1^ for the lag phase.

For *A. carbonarius* with the use of oregano EH, the growth rate values ranged from 7.2 to 9.8 mm day^−1^ and the lag phase was between 16 and 19 h (Table 7). For the growth rate values, only the 50 and 600 mg kg^−1^ concentrations were statistically different to the control (*p* < 0.05), although there was no statistically significant difference between the lag phase values.

The use of clove ethanolic extract on *A. westerdijkiae* resulted in a growth rate ranging from 0.5 to 6.2 mm day^−1^ and the lag phase was between 32 and 137 h (Table 8). All the concentrations were statistically different to the control considering the growth rate, although, for the lag phase only 300 and 600 mg kg^−1^ showed statistical differences on *A. westerdijkiae* (*p* < 0.05).

The growth rate values of *A. carbonarius* ranged from 0.5 to 9.8 mm day^−1^ and the lag phase ranged between 19 and 130 h with clove ethanolic extract (Table 9). Only the concentrations 100, 300, and 600 mg kg^−1^ were statistically different to the control for growth rate, and, for the lag phase, only the two higher concentrations (300 and 600 mg kg^−1^) were effective.

## 3. Discussion

The hexanic plant extracts showed the best effects by leading to a complete inhibition against both *Aspergillus* strains tested. On the other hand, the initial screening with hydrodistilled essential oils showed that the best inhibitory effect occurred with the 2.5 μL mL^−1^ of cinnamon EO. El Khoury et al. (2016) [40] demonstrated a complete inhibition by using a 2-fold concentration (5 μL mL^−1^) of cinnamon EO against the growth of *A. carbonarius* [40], a result consistent with this study. Other papers had demonstrated lower concentrations necessary to inhibit the *A. carbonarius* growth, e.g., the inhibition using 1.67 μL mL^−1^ and using 50 μL L^−1^. However, these different results could be possibly due to the extreme differences in the methodology used, such as the medium or the concentration of the spore inoculum or even the composition of the cinnamon oil [26,40,41,42,43].

Despite the great inhibitory effect of the cinnamon EO, the low productivity of the oil precluded the evaluation of the lag phase and the growth rate. Therefore, even though cinnamon EO showed a great activity the oils and extracts chosen to be analyzed were rosemary and oregano hydrodistilled essential oils and oregano and clove ethanolic extract.

The rosemary EO significantly reduced the *A. carbonarius* growth rate at all concentrations tested; however, the lag phase was also significantly reduced, an opposite result of what was expected. The effect of rosemary EO on growth of *A. westerdijkiae* led to a reduction only at the lowest concentration, 100 mg kg^−1^. These results have a high impact and novelty due to the lack of papers, using rosemary EO as an antifungal agent against these two *Aspergillus* species, and the differences in the methodology among the few previous studies. Another important fact that could elucidate the disagreement between these results and other papers is that *Rosmarinus officinalis* L. from distinct origins can present different secondary metabolite profiles. The rosemary EO can exhibit distinguishable physicochemical characteristics and different major components. This plant has already been classified in five chemotypes [45,46,47]. A previous study had showed that 1 μL mL^−1^ and 5 μL mL^−1^ of rosemary EO resulted in a 11% reduction in *A. carbonarius* growth, which is a similar result to the one found in the initial screening test using 2.5 μL mL^−1^ [40]. Despite the innumerous research on rosemary essential oils, none of these studied the effects of this oil against the fungal growth of *A. westerdijkiae*. The main papers published about this EO reported the antimicrobial effect of this oil against other *Aspergillus* species, such as *A. flavus*, *A. parasiticus*, *A. niger*, or bacteria and pathogenic fungi [18,23,24,26,27,29,30,31,32,33,34,35,36,37,38]. Meanwhile, the lag phases that had significant differences to the control exhibited a reduced time, this result being different to what was expected.

The growth rate for both strains on the five concentrations of oregano EO obtained using hydrodistillation had a significant reduction compared to the control; the greatest growth rate inhibition was 600 mg kg^−1^ for *A. westerdijkiae* and 300 mg kg^−1^ for *A. carbonarius*. The results for the lag phase were also as expected, with a significant increase in the hours, being an increase of 55 h for *A. westerdijkiae* at 600 mg kg^−1^ and 20 h for *A. carbonarius* at 300 mg kg^−1^. These results were divergent to another study that had detected a complete inhibition of oregano EO against *A. carbonarius* at 1 and 5 μL mL^−1^, however, the methodology was different regarding the culture media used, Synthetic Grape Medium (SGM), and incubation time of only 4 days, which could explain the differences found between both papers [40]. Other research has detected a similar inhibition when using 500 ppm of oregano EO against the growth of *A. westerdijkiae* and identified a complete inhibition at higher concentrations of 750 and 1000 ppm [39]. Another possible explanation for the divergent results found between the research is the variability in the essential oil compositions. There are four different chemotypes of *Origanum vulgare* L. already described, and the main two are carvacrol and thymol types [48,49,50].

The antifungal activity of oregano EO against other fungi genera, *Fusarium* spp. and *Aspergillus* spp. strains, such *A. niger*, *A. parasiticus*, and *A. flavus*, has been examined. However, due to the differences in the species tested, the results are completely different from the ones described in this study [16,18,23,24,25,26,27,28,29,30,31,32,33,34,35,36,37,38].

In contrast to the great results found for oregano hydrodistilled EO activity, the oregano hexanic plant extract (EH) reduced the growth rate and increased the lag phase only for *A. westerdijkiae* at 150 mg kg^−1^. The oregano EH showed a low inhibition activity against the growth of both tested strains, demonstrating that the hydrodistillation method is highly efficient in obtaining oregano essential oil with antifungal activity. This result agreed with another study that showed a worse antifungal activity of the oregano extract than the oregano essential oil [51].

The clove ethanolic extract showed the best results from all essential oils and extracts tested. The concentration of 300 and 600 mg kg^−1^ showed an outstanding performance on both strains assayed. For *A. westerdijkiae* at concentrations of 300 and 600 mg kg^−1^, the lag phase was extended to 125 and 137 h. This represents a lag phase extension of at least five days, which shows the incredible activity of this extract. In addition, the growth rate was reduced from 6.2 to 1.5 and 0.5 mm day^−1^, respectively. This extract also showed a high performance with the *A. carbonarius* strain, at the concentrations of 300 and 600 mg kg^−1^ the lag phase was prolonged to 114 and 130 h, respectively, which means a lag phase of at least 4.75 days. Besides that, the growth rate was reduced from 9.8 to 0.6 and 0.5 mm day^−1^. The good activity of clove EE and EO found in this study and others might be due to the major compound eugenol [35]. These results could not be compared with other articles, since the papers which had studied the antifungal effects of clove oil used different *Aspergillus* species or even other fungal genera; this fact demonstrates the relevance and novelty of this research.

The clove extract was proved to have a good antifungal activity against other microorganisms, for example, the concentration needed to inhibit 87% of *A. flavus* was similar to this study, 500 mg kg^−1^ [36]. In addition, this same concentration was proved to totally inhibit the growth of *Fusarium oxysporum*. In another article, however, for the inhibition of *A. niger* only 400 mg kg^−1^ was needed [27]. Clove extract has also been known to have an important role in mold control against *A. parasiticus* and in aflatoxin prevention, but this study showed that other extracts of clove, obtained using different methods of extraction, have antifungal activity against some *Aspergillus* species.

Despite that, this paper proved that the clove ethanolic extract would be useful to control fungal growth in food at concentrations higher than 300 mg kg^−1^; the activity of these oils and extracts against mycotoxin production was not yet evaluated. Rodrigues et al. (2019) [52] showed that higher concentrations of neem essential oil could increase the ochratoxin A (OTA) production [52], therefore, future studies will be conducted to clarify the effects of extracts and essential oils on the production of OTA.

## 4. Materials and Methods

### 4.1. Fungal Strains 

The referenced strains used were *Aspergillus westerdijkiae* NRRL 3174 and *Aspergillus carbonarius* RC 2054.

### 4.2. Plant Materials

Plant species used in this work were basil (*Ocimum basilicum*), cinnamon (*Cinnamomum zeylanicum*), clove (*Eugenia caryophyllata*), cumin (*Cuminum cyminum*), marjoram (*Origanum majorana*), nutmeg (*Myristica fragrans*), oregano (*Origanum vulgare*), rosemary (*Rosmarinus officinalis*), spearmint (*Menta piperita*), and sweet fennel (*Pimpinella anisum*). All plant material were kindly provided by Vitalis Indústria de Alimentos, Ltda. (Rio de Janeiro, Brazil).

### 4.3. Essential Oils

The essential oils were obtained from all 10 plants using hydrodistillation, which was carried out in a conventional Clevenger-type distillation apparatus in which the plant material to water ratio was 100 g of plant material for 1 L of water for 1 h. After steam distillation, the essential oil (EO) samples were dried with anhydrous sodium sulfate and stored in sterilized vials at 4 °C until use, protected from light [53]. The total income of every oil produced was properly calculated. Appropriate measures to eliminate photolysis transformations and volatilization losses were applied, including wrapping glassware in aluminum foil; using a borosilicate amber glass vial sealed with Teflon-faced septum and plastic screw cap; and performing all analyses within 30 days.

### 4.4. Plant Extracts

The aqueous extract (AE) was obtained from the aqueous phase resulting from the hydrodistillation process for the 10 herbs [54]. The ethanolic extraction (EE) was completed according to Thanaboripat et al. [29], passively and under dark conditions, using a 1:2 *w*/*v* (material/solvent) ratio for 48 h. The extract was filtered and concentrated in a rotary evaporator. The hexanic extraction was adapted from Kuiate et al. [55] with a proportion of plant material/solvent 1:2 *w*/*v*, in the dark and passively for 48 h. The obtained filtrate was evaporated in a water bath at 80 °C to obtain a quantity of 10 mL of each hexanic plant extract (EH). All vegetal extracts were stored in amber sterilized bottles under refrigeration at about 4 °C until use.

### 4.5. Initial Screening

A total of 10 essential oils and 30 plant extracts were tested using the diffusion agar method. A fungal spore suspension (10^6^ conidia per mL), adjusted with the Neubauer chamber, was incorporated into 20 mL of yeast extract sucrose agar (YES) and then poured into Petri dishes. After the agar solidification, five equidistant wells were made using a punch. Then, 50 μL of EO and PE of each plant was pipetted into the wells. Each assay was performed in triplicates. The inhibition ring of fungal growth was evaluated using metric comparison.

### 4.6. Effect of the Essential Oils and Vegetal Extracts on Growth Parameters

The EO or plant extracts that demonstrated good efficiency in inhibiting fungal growth in the screening test were tested to obtain the growth parameters. First, they were added to 20 mL of YES at about 45–50 °C at the concentrations of 0, 50, 100, 150, 300, and 600 mg kg^−1^. Then, the toxigenic strains were centrally inoculated using a needle-shaped sterile loop, with fungi spores from a suspension in semi-solid agar with Tween^®^ 80 (approximately 10^3^ conidia per inoculation). The Petri dishes were incubated at 25 °C for the maximum of two weeks and the fungal growth was examined daily by measuring the diameter of the growing fungal colonies [52]. All assays were prepared in triplicates.

### 4.7. Statistical Analyses

An analysis of variance (ANOVA) was used for lag phase and growth rate using the SAS program (SAS Institute Inc, Cary, NC, USA). The treatments mean values were compared using the Dunnett test at the *p* < 0.05 level. This test controls the Type I experiment error for comparisons of all treatments against the control.

## 5. Conclusions

This study proved that some essential oils and plant extracts have good antifungal activity against *Aspergillus carbonarius* and *A. westerdijkiae*, both ochratoxin A producers, and that by increasing the concentrations of the plant oils and/or extracts the antimicrobial effect enhances. However, further studies must be conducted to clarify the effects of extracts and essential oils on mycotoxin production.

## Figures and Tables

**Table 1:** The inhibitory effect of essential oils and extracts on *Aspergillus westerdijkiae* growth using the measurement of the diameter of inhibition zone in mm using the agar diffusion method.

Plants	EO *	EH	EE *	AE
Rosemary	16 ± 3.91	CP ^a^	15 ± 3.59	NO
Cinnamon	60 ^a^ ± 2.86	CP ^a^	NO	NO
Cumin	NO	CP ^a^	12 ^a^ ± 2.91	NO
Clove	NO	CP ^a^	55 ± 4.89	NO
Sweet fennel	26 ^a^ ± 1.81	CP ^a^	28 ^a^ ± 2.37	NO
Spearmint	NO	CP ^a^	NO	NO
Basil	NO	CP ^a^	NO	NO
Marjoram	NO	CP ^a^	NO	NO
Nutmeg	NO	CP ^a^	16 ^a^ ± 4.14	NO
Oregano	38 ± 1.49	CP	NO	NO

* Mean values ± standard deviation based on triplicated data; ^a^ the income from oil production was inefficient for further tests; CP = complete inhibition; NO = inhibitory effect not observed.

**Table 2:** The inhibitory effect of essential oils and extracts on *Aspergillus carbonarius* growth using the measurement of the diameter of inhibition zone in mm using the agar diffusion method.

Plants	EO *	EH	EE *	AE
Rosemary	15 ± 2.49	CP ^a^	NO	NO
Cinnamon	55 ^a^ ± 0.46	CP ^a^	NO	NO
Cumin	NO	CP ^a^	12 ^a^ ± 0.44	NO
Clove	NO	CP ^a^	46 ± 3.15	NO
Sweet fennel	26 ^a^ ± 3.60	CP ^a^	NO	NO
Spearmint	NO	CP ^a^	NO	NO
Basil	NO	CP ^a^	NO	NO
Marjoram	NO	CP ^a^	NO	NO
Nutmeg	NO	CP ^a^	10 ^a^ ± 0.46	NO
Oregano	42 ± 1.45	CP	NO	NO

* Mean values ± standard deviation based on triplicated data; ^a^ the income from oil production was inefficient for further tests. CP = complete inhibition; NO = inhibitory effect not observed.

**Table 3:** Effect of rosemary essential oil on the lag phase and growth rate of *A. westerdijkiae* at different concentrations.

Rosemary EO			
Strain	mg kg^−1^	Growth Rate (mm day^−1^)	Lag Phase (h)
*A. westerdijkiae*	0	6.0 ± 0.10	31 ± 0.78
	50	6.0 ± 0.19	28 * ± 1.49
	100	5.3 * ± 0.14	25 * ± 1.07
	150	6.0 ± 0.01	27 * ± 1.73
	300	6.1 ± 0.10	29 * ± 0.54
	600	6.0 ± 0.06	31 ± 1.59

* Mean values ± standard deviation, based on triplicated data, with significant differences according to the Dunnett test (*p* < 0.05).

**Table 4:** Effect of rosemary essential oil on lag phase and growth rate of *A. carbonarius* at different concentrations.

Rosemary EO			
Strain	mg kg^−1^	Growth Rate (mm day^−1^)	Lag Phase (h)
*A. carbonarius*	0	10.8 ± 0.01	28 ± 0.16
	50	10.2 * ± 0.07	26 * ± 0.11
	100	10.1 * ± 0.21	26 * ± 1.80
	150	10.0 * ± 0.20	25 * ± 1.17
	300	10.6 * ± 0.18	25 * ± 2.20
	600	10.4 * ± 0.21	26 * ± 0.87

* Mean values ± standard deviation, based on triplicated data, with significant differences according to the Dunnett test (*p* < 0.05).

**Table 5:** Effect of oregano essential oil on lag phase and growth rate of *A. westerdijkiae* and *A. carbonarius* at different concentrations.

Oregano EO			
Strain	mg kg^−1^	Growth Rate (mm day^−1^)	Lag Phase (h)
*A. westerdijkiae*	0	5.6 ± 0.10	33 ± 0.78
	50	4.0 * ± 0.05	41 * ± 1.29
	100	3.6 * ± 0.18	55 * ± 0.76
	150	5.0 * ± 0.06	37 ± 1.16
	300	3.3 * ± 0.20	59 * ± 0.77
	600	1.3 * ± 0.47	88 * ± 1.13
*A. carbonarius*	0	11.0 ± 0.04	33 ± 0.16
	50	5.4 * ± 0.74	39 * ± 0.19
	100	3.0 * ± 0.55	50 * ± 0.02
	150	4.4 * ± 0.32	43 * ± 0.81
	300	2.8 * ± 0.07	53 * ± 0.30
	600	3.3 * ± 0.35	48 * ± 0.03

* Mean values ± standard deviation, based on triplicated data, with significant differences according to the Dunnett test (*p* < 0.05).

**Table 6:** Effect of oregano hexanic plant extraction on lag phase and growth rate of *A. westerdijkiae* at different concentrations.

Oregano EH			
Strain	mg kg^−1^	Growth Rate (mm day^−1^)	Lag Phase (h)
*A. westerdijkiae*	0	6.2 ± 0.05	32 ± 0.40
	50	6.1 ± 0.01	32 ± 0.07
	100	5.7 ± 0.69	30 ± 3.78
	150	5.3 * ± 0.04	27 * ± 1.85
	300	5.5 * ± 0.37	29 ± 2.89
	600	6.2 ± 0.24	35 ± 0.72

* Mean values ± standard deviation, based on triplicated data, with significant differences according to the Dunnett test (*p* < 0.05).

**Table 7:** Effect of oregano hexanic plant extraction on lag phase and growth rate of *A. carbonarius* at different concentrations.

Oregano EH			
Strain	mg kg^−1^	Growth Rate (mm day^−1^)	Lag Phase (h)
*A. carbonarius*	0	9.8 ± 0.01	19 ± 0.40
	50	7.3 * ± 0.30	17 ± 1.43
	100	8.1 ± 0.31	16 ± 0.80
	150	9.5 ± 0.14	17 ± 1.19
	300	9.7 ± 0.24	16 ± 1.57
	600	7.2 * ± 0.39	19 ± 0.75

* Mean values ± standard deviation, based on triplicated data, with significant differences according to the Dunnett test (*p* < 0.05).

**Table 8:** Effect of clove ethanolic extract on lag phase and growth rate of *A. westerdijkiae* at different concentrations.

Clove EE			
Strain	mg kg^−1^	Growth Rate (mm day^−1^)	Lag Phase (h)
*A. westerdijkiae*	0	6.2 ± 0.05	32 ± 0.40
	50	2.7 * ± 0.02	45 ± 2.32
	100	3.7 * ± 0.07	40 ± 1.22
	150	1.5 * ± 0.12	65 ± 2.67
	300	1.5 * ± 0.25	125 * ± 2.13
	600	0.5 * ± 0.18	137 * ± 0.33

* Mean values ± standard deviation, based on triplicated data, with significant differences according to the Dunnett test (*p* < 0.05).

**Table 9:** Effect of clove ethanolic extract on lag phase and growth rate of *A. carbonarius* at different concentrations.

Clove EE			
Strain	mg kg^−1^	Growth Rate (mm day^−1^)	Lag Phase (h)
*A. carbonarius*	0	9.8 ± 0.04	19 ± 0.33
	50	5.6 ± 0.02	19 ± 0.24
	100	4.0 * ± 0.12	34 ± 0.77
	150	5.1 ± 0.26	21 ± 0.03
	300	0.6 * ± 0.14	114 * ± 0.96
	600	0.5 * ± 0.10	130 * ± 0.01

* Mean values ± standard deviation, based on triplicated data, with significant differences according to the Dunnett test (*p* < 0.05).

## Data Availability

Not applicable.
